# Characteristics of cutaneous adverse drug reactions with special respect to the incubation period based on hospitalized patients^☆^

**DOI:** 10.1016/j.abd.2022.05.003

**Published:** 2023-01-25

**Authors:** Xiaoli Chen, Li Hu, Zupeng Xiao, Hanyi Wu, Aijun Chen, Rentao Yu

**Affiliations:** Department of Dermatology, The First Affiliated Hospital of Chongqing Medical University, Chongqing, China

Dear Editor,

More medication choices, extended treatment courses, and longer patient survival contribute to increased exposure to drugs and subsequently give rise to the incidence of Cutaneous adverse drug reactions (CADRs).^1^, ^2^, ^3^ Although most patients with CADRs will be cured after drug withdrawal, severe types of CADR require hospital interventions and are even life-threatening.^4^, ^5^ This report retrospectively analyzed the characteristics of patients with CADRs based on hospitalized patients. Specifically, we focused on the Incubation period (IP) and associated factors.

A total of 308 confirmed patients with CADRs from 2013 to 2018 hospitalized at the First Affiliated Hospital of Chongqing Medical University were enrolled in this study. The demographic and clinical characteristics of these patients were collected from the electronic medical system. The relationship between IPs and other factors was analyzed by correlation analysis, and the differences in levels of IPs among different subgroups were compared by the Kruskal-Wallis test. This study was reviewed and approved by the Ethics Committee of the First Affiliated Hospital of Chongqing Medical University.

Table 1 showed the baseline characteristics of enrolled patients. The median age of enrolled patients was 47 yrs with 49.7% females. The results showed that erythema multiforme and maculopapular exanthema were the most common types, accounting for 30.5% and 26.6%, respectively. Severe CADRs like Steven-johnsons syndrome/Toxic epidermal necrolysis (SJS/TEN) and Drug reaction with eosinophilia and systemic symptoms (DRESS) covered 21% of them. The median IP was 4 days with a median length of stay of 7 days. Besides, skin lesions in about 40% of patients the mucosa was affected and over 70% of patients developed CADRs by means of oral administration. Regarding the culprit drugs, antibiotics were the most common drugs, covering 36.7% of all patients, followed by Chinses herbs (24.7%), non-steroidal anti-inflammatory drugs (10.1%), and anticonvulsants (8.8%). Furthermore, we compared the types of culprit drugs between patients with severe and mild-to-moderate CADRs. The result showed a significant difference between the two subgroups (p < 0.001). Anticonvulsants and allopurinol seemed associated with severe types of CADRs (Fig. 1).

**Table 1:** Clinical characteristics of patients with cutaneous adverse drug eruptions.

	All patients (n = 308)
**Age, years**	47.0 (31.0‒62.0)
**Gender, female**	153 (49.7%)
**Diagnosis**	
SJS/TEN	47 (15.3%)
DRESS	17 (5.5%)
Erythema multiforme	94 (30.5%)
MPE	82 (26.6%)
Fixed drug eruption	54 (17.5%)
Others	14 (4.5%)
**Incubation period, days**	4.0 (1.0‒10.0)
IP of DRESS	20.0 (4.0‒28.5)
**Mucosa involvement**	128 (41.6%)
**Length of stay, days**	7.0 (5.0‒11.0)
**Comorbidities**	
Hypertension	50 (16.2%)
Diabetes	46 (14.9%)
Chronic renal disease	14 (4.5%)
Cancer	10 (3.2%)
COPD	7 (2.3%)
Autoimmune disease	20 (6.5%)
Epilepsy	16 (5.2%)
**Culprit drug**	
Antibiotics	113 (36.7%)
Chinese herbs^b^	76 (24.7%)
NSAIDS^c^	31 (10.1%)
Anticonvulsants^d^	27 (8.8%)
Allopurinol	8 (2.6%)
Anticancer drugs	5 (1.6%)
Anti-virus drugs	3 (1.0%)
Others	24 (2.9%)
Unknown drugs	21 (6.8%)
**Route of administration**	
Oral	223 (72.4%)
Intramuscular injection	2 (0.6%)
Intravenous injection	62 (20.1%)
Unknown	21 (6.8%)
**Treatment**	
Antihistamines alone	11 (3.6%)
Cyclosporine alone	2 (0.6%)
GC alone	266 (86.4%)
GC + Azathioprine	2 (0.6%)
GC + Cyclosporine	9 (2.9%)
GC + IVIG	3 (1.0%)
Glycyrrhizin alone	9 (2.9%)
Methotrexate	1 (0.3%)
Only supportive care	5 (1.6%)

COPD, Chronic Obstructive Pulmonary Disease; DRESS, Drug Reaction with Eosinophilia and Systemic Symptoms; GC, Glucocorticoids; IVIG, Intravenous Immunoglobulin; MPE, Maculopapular Exanthema; NSAIDS, Non-Steroidal Anti-Inflammatory Drugs; SJS, Stevens-Johnson Syndrome; TEN, Toxic Epidermal Necrolysis.

^a^Antibiotics include penicillins, macrolides, quinolones, cephalosporins, sulfonamides, aminoglycosides and miscellaneous antibiotics.

b Chinese herbs include Chinese patent drugs, herbal slice and self-made herb.

c NSAIDS include non-Selective NSAIDS like aspirin and ibuprofen, and selective NSAIDS like celecoxib and meloxicam.

d Anticonvulsants include carbamazepine, phenytoin, lamotrigine and gabapentin.

**Figure 1:**
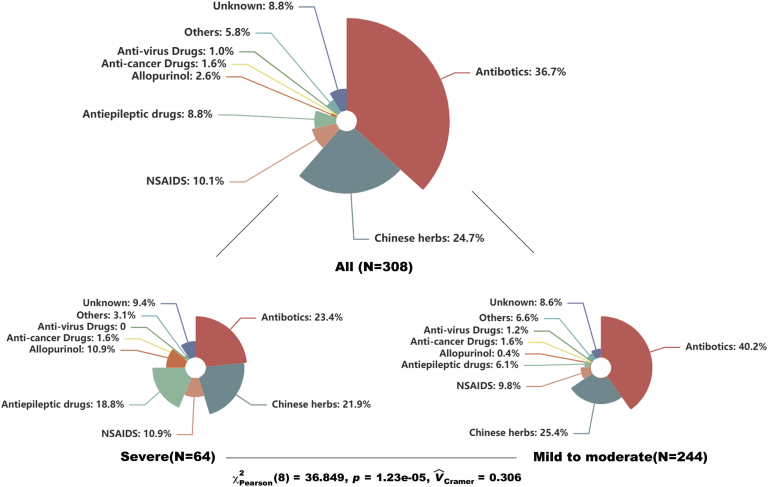
Distributions of types of culprit drugs among all patients, and patients of severe and mild-to-moderate types of cutaneous adverse drug reactions. The difference was compared using the Chi-square test and Cramer’s V was calculated. NSAIDS, Non-Steroidal Anti-Inflammatory Drugs.

IP was the priority of this report, and we further analyzed factors associated with IP, as shown in Table 2. The result showed that disease types (χ^2^ = 0.153, p < 0.001), route of administration (χ^2^ = 0.060, p < 0.001), and culprit drugs (χ^2^ = 0.151, p < 0.001) were the significantly correlated factors. However, no significant association could be detected between IPs and gender (r_rank-biserial_ = 0.002, p = 0.973), age (rho = 0.104, p = 0.068), and mucosa involvement (r_rank-biserial_ = 0.132, p = 0.054). Based on the results above, we then compared levels of IP among different subgroups (Fig. 2). Patients administered allopurinol and anticonvulsants had longer IPs than other drugs, oral administration longer than the injection, and severe CADRs longer than mild-to-moderate CADRs (p < 0.05 for all, Fig. 2A‒C). The same association could also be seen if we presented the proportion of each subgroup by IP every 3 days (Fig. 2D‒F). The figure showed that although almost every subgroup could be detected in each group categorized by IP, the distribution of each subgroup was skewed and accumulated in certain IP categories, indicating the association between IP and these factors.

**Table 2:** Factors associated with incubation period.

	Effect size	p	Measures
Gender	0.002	0.973	Rank-Biserial
Age	0.104	0.068	Spearman’s rho
Disease types	0.153	<0.001	χ^2^
Route of administration	0.060	<0.001	χ^2^
Mucosa involved	0.132	0.054	Rank-Biserial
culprit drug	0.151	<0.001	χ^2^

**Figure 2:**
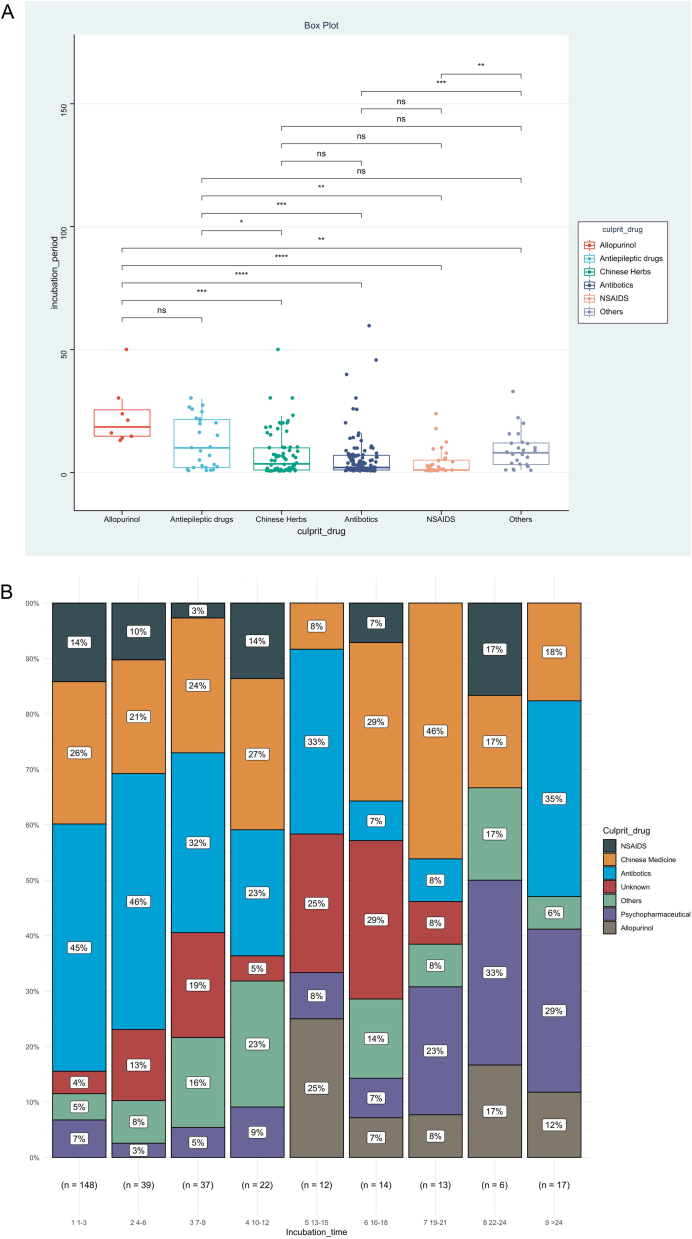

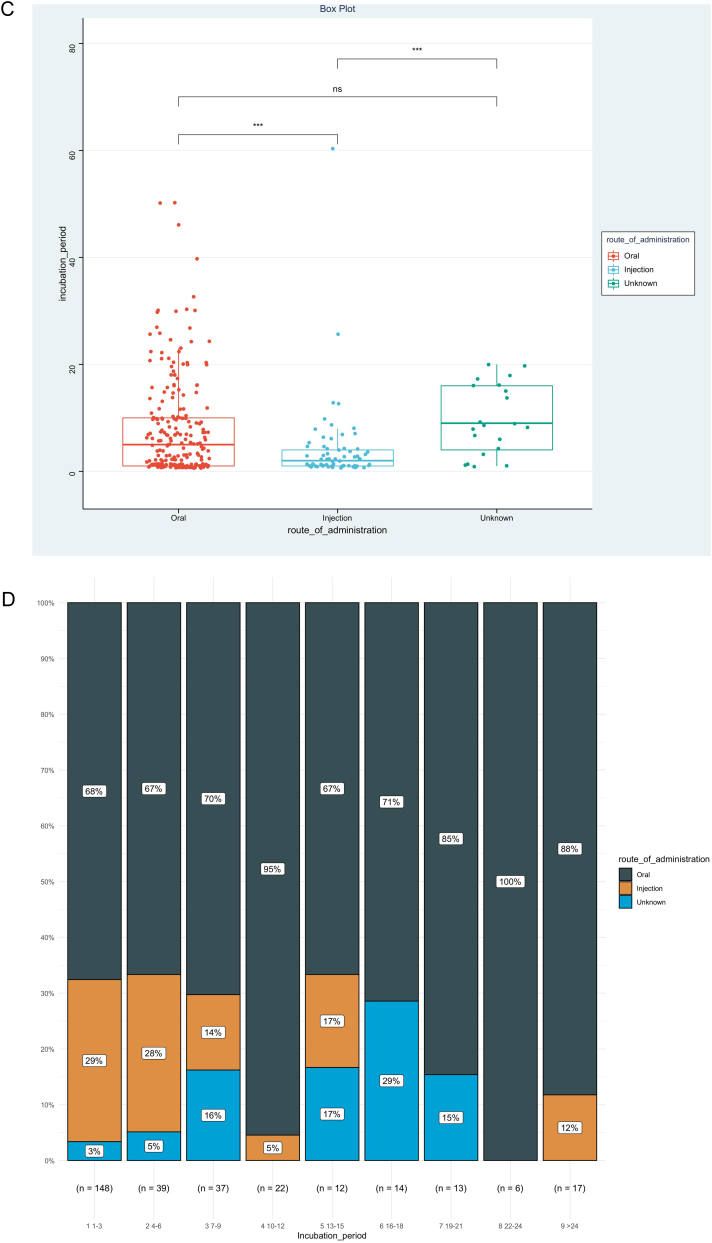

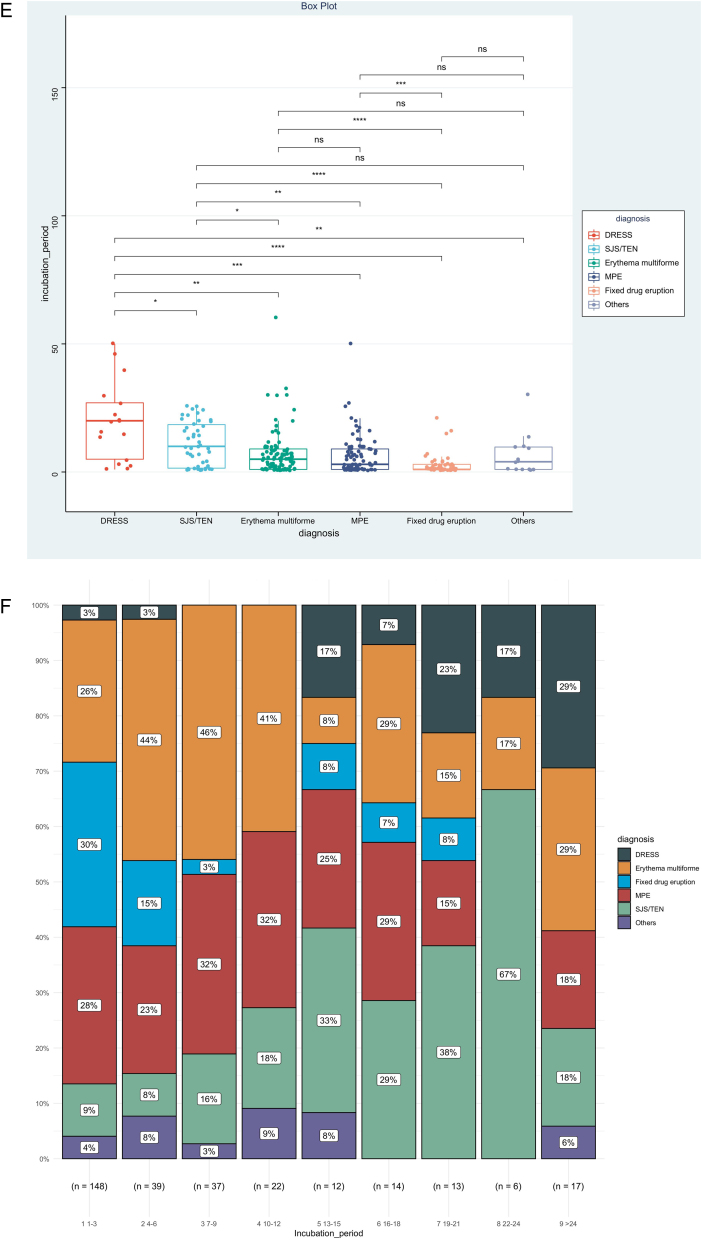
Levels and distributions of incubation period among subgroups of patients with cutaneous adverse drug reactions. The levels of the incubation period were compared using the Kruskal-Wallis test and the post hoc analysis was adjusted using the Bonferroni test. DRESS, Drug reaction with eosinophilia and systemic symptoms; MPE, Maculopapular exanthema; NSAIDS, Non-steroidal anti-inflammatory drugs; SJS, Stevens-johnson syndrome; TEN, Toxic epidermal necrolysis.

This study thoroughly analyzed the IPs of hospitalization-based CADRs and associated factors. However, the main limitation of this study is how to precisely determine the culprit drugs and IPs. Although IP and the culprit drug of each CADR patient were recorded in the electronic medical system, the nature of the retrospective design implied that the criteria were not unified. Consequently, the results may be biased.

In conclusion, this descriptive analysis suggested that severe and mild-to-moderate types of CADRs might be different diseases, especially in culprit drugs and IPs. Longer IPs were significantly associated with severe types, oral administration and allopurinol/anticonvulsants. This result may be helpful in understanding the IPs of CADRs and assessing the severity of CADRs.

## Financial support

This study was supported by the Postdoctoral Research Foundation of Chongqing Medical University (nº 2-01-02-04-P0474) and the Special Foundation for Postdoctoral Research Projects of Chongqing, Grant Number: 2021XM3080.

## Authors’ contributions

Xiaoli Chen: Methodology; data curation; visualization.

Li Hu: Conceptualization; project administration.

Zupeng Xiao: Resources; writing - review & editing.

Hanyi Wu: Validation; data curation.

Aijun Chen: Investigation; supervision.

Rentao Yu: Formal analysis; funding acquisition; software; roles/writing - original draft.

## Conflicts of interest

None declared.
